# Psychological support and strategies for raising motivation in fresh students

**DOI:** 10.3389/fpsyg.2022.1019653

**Published:** 2023-01-19

**Authors:** Tao Li, Vitaly Pichugin, Irina Shaknmalova, Nailya Ismailova, Svetlana Salimova

**Affiliations:** ^1^Organization Department, Tonghua Normal University, Tonghua, China; ^2^Department of HR Management and Psychology, Financial University under the Government of the Russian Federation, Moscow, Russia; ^3^Department of Pedagogy and Methods of Primary Education, Nerungri Technical Institute (Branch) of North-Eastern Federal University, Neryungri, Russia; ^4^Department of Psychology, Yelabuga Institute of Kazan Federal University, Yelabuga, Russia; ^5^Department of English Language for Professional Purposes, Ogarev Mordovia State University, Saransk, Russia

**Keywords:** motivation, novice students, coping fears, psychological distress, higher education

## Abstract

The goal of this study was to investigate the of fear of starting university training, determine its general characteristics, and test how providing psychological and motivational support for university enrollees may influence the elimination of this type of fear. The study used a random sample of 536 individuals, 268 participants each from one university in the Russian Federation and one from China, who were also randomly selected. Approximately equivalent experimental and control groups (134 people) were formed. The main experiment was conducted simultaneously for Chinese and Russian groups of participants under the same conditions. The modified Kessler psychological distress scale (K10) was used to measure fear of starting training 10 days before training and 2 days after. The experimental group received a special 7-step training to overcome fear and prepare for learning; results between groups and results before and after the start of training were compared using Student’s *t*-test. Test scores for each participant were tested using Pearson’s correlation to establish an association with the intervention for the majority of participants. In sum, the results of the carried out testing demonstrate a marked reduction in fear and stress after the start of training for both groups of both universities. Those students who have already experienced support show lower levels of fear of starting a course than those who have not received any help. The results of this study can be used to prepare university applicants for their future studies.

## Introduction

The study of fears is one of the most important areas of research in modern pedagogy and psychology (Frolova et al., 2021). In many cases, a number of chronic fears and anxieties that emerged and solidified in the school age continue to have devastating effects on the individual during college or university study (Jimenez et al., 2010). Anxiety has been studied fruitfully for a long time in the context of student behavior; fears in the learning process have been studied to a lesser extent (Shoyer and Leshem, 2016). In the context of this study and in accordance with generally accepted practice, fear is hereinafter defined as a stronger emotional experience associated with inhibition of activity and activation of the parasympathetic nervous system, which, with significant forces, can paralyze human activity (Mineka and Sutton, 2006). Anxiety is associated with general arousal of a person, in particular, the sympathetic nervous system and is associated with more indefinite stimuli, may occur in the absence of a real assessed danger (Mineka and Zinbarg, 2006).

In the predominance of cases, students who experience anxiety or worry of any etiology are able to cope with the problem either on their own or with the help of friends, family, or qualified professionals (Sunderland et al., 2021). In light of this, many educational institutions use specific techniques to support and accompany learners thus reducing their exposure to various types of stressors and promoting fear reduction (Ginat-Frolich et al., 2017). At the same time, according to the data available, such programs have not gained compulsory and all-encompassing prevalence these days (Shoyer and Leshem, 2016).

The nature of fear has been studied for a long time. As a result, a large number of individual fears and clinical phobias were identified through a consistent investigation of various fear behaviors and associated reactions (Taylor, 1998; Mineka and Sutton, 2006). Scholars have made numerous attempts to create a workable, principles-based classification of fears and provide definitions suitable for use outside of the clinical application. However, a major difficulty here is that the model of fear used in clinical care for patients with mental disorders is rarely adequate for practical psychology and pedagogy due to dramatically different objectives (Guerrero, 2007). In particular, physiological technical laboratory tests that allow for a more accurate patient diagnosis are neither possible nor necessary in a school or university. Determining the nature, predictors, and characteristic manifestations of certain fears in the field of education is most often done by the subjective assessment of learners or by external observation of their behavior (Patel and Dhar, 2020). In this case, the serious problem is that researchers often can determine neither the origin of feelings of fear or anxiety nor their relative strength (Mineka and Zinbarg, 2006).

Academic world representatives are likely to identify several major fears that are consistently evident in high school and college students and are closely related to the learning process (Jackson, 2017; Hasan and Bao, 2020). These are fear of failure, fear of public speaking, fear of social unrecognition, and fear of a test. In these latter days, increasing attention has also been directed toward the fear of success, which can act as a form of reaction to disruption of an individual’s routine or order of things (Henriksen et al., 2020). Among other forms of fear of learning that are not always recognized is also the fear of starting something new, which often manifests in preschool and school-age children, predominantly in connection with specific stresses, homeschooling, or the way of living (Michalska et al., 2016).

A crucial aspect of the study of learning-related fears is analyzing the memory mechanisms of the experienced fear or situations that led to the formation of a sustained fear as a response to a stressor stimulus (Keckler, 2018). By and large, the situations that caused fears of learning are not directly related to schooling, but most fears experienced by college and university students originate from school time (Jimenez et al., 2010). Therefore, researchers strongly emphasize the importance of external help, psychological support, and building sustained motivation to learn in school and college students since, as they note, at some level of stressor accumulation, it is nearly impossible for individuals to cope with fear on their own (Li and Morris, 2007; Henriksen et al., 2020).

### Literature review

Fear research is closely related to studying the structure and classification of different types of fear and their relationship. Unfortunately, most works on this issue were published more than 10 years ago and continue to be a reference for contemporary research on fear classification (Dias et al., 2016; Ginat-Frolich et al., 2017; Patel and Dhar, 2020). These studies rely on categorizing fears into groups based on their relevance to the underlying needs or fundamental dangers an individual responds to. All of them tend to recognize the fear of death as one of the fundamental ones joined by fears of various sources of physical danger, which can be both real and contrived (fear of spiders, snakes, big beasts; fear of physical mutilation, etc.). Such a hierarchy assumes a number of fears that are associated with social relations to be of a higher level of organization. These refer, in particular, to the fear of no social recognition, fear of loneliness, fear of crowds, and complementary fears of success and failure. Closely related to these is the fear of testing or fear of being tested, recognized by many scholars (Bell and Williams, 2006; Chetty and van der Westhuizen, 2013). Almost all of the varieties of fear described are, to some extent, characteristic of fears that arise during or before learning (Conrad, 2002; Alt, 2015; Corral-Lage et al., 2020).

Observations of children’s behaviors, particularly those of preschool age, demonstrate that a set of learning-related fears is present in them even before learning begins (Correia and Marques-Pinto, 2016; Carneiro et al., 2018). These behaviors are also demonstrated throughout high school and during university (Taylor, 1998; Garcia-Ruffin, 2003; Frolova et al., 2021). Empirical evidence supports the persistence of this tendency for fears before and during tertiary education (post-secondary and continuing education or professional development) (Rogerson and Scott, 2010).

Scientific works on the topic suggest that isolated study of separate fears in a context as complex as academic learning is ineffective because individual components of experiences can be challenging to separate from background experiences or stereotypical facts about the psychological life of the person being analyzed (Mineka and Sutton, 2006; Jackson, 2017). In almost all cases, researchers are dealing with a set of relatively persistent experiences that may follow similar scenarios, which actually allows them to be conventionally identified and terminologically described as one or another fear (Li and Morris, 2007; Jimenez et al., 2010; Keckler, 2018). However, it should be understood that in each case, such a “fear” is more of an academic construct than a precisely defined medical or neurophysiological fact that is convenient and adequate for practical purposes, e.g., to cure fear and anxiety during learning.

An in-depth study of fears directly related to learning and their conquering methods allows revealing a number of their common features and thus enables more precise identification of the phenomenon that this paper is devoted to. The practice shows that in different circumstances and learning contexts, there is an intensification of the strength of fear and anxiety immediately in a period preceding the beginning of learning. And what is more important, some people cannot cope with the pressure of fear, which can act as a predictor of academic failure and even early withdrawal from learning (Pleše et al., 2018). The current work proposes considering this phenomenon as a complex psychological state that includes fears and experiences related to learning and calling it fear of starting training.

Most academic papers in pedagogy or educational psychology have focused on particular types of fears or concerns and their impact on academic success, well-being, and student behavior (Hasan and Bao, 2020). Nevertheless, there is a significant gap in research on fears that operate before the start of university training and then continue to persist or arouse during the learning process. That said, this type of fear, as shown by existing studies, has unique characteristics that set it apart from other types of stress (Mineka and Zinbarg, 2006; Shoyer and Leshem, 2016; Patel and Dhar, 2020).

The ultimate goal of this study was to investigate the phenomenon of fear of starting university training, determine its general characteristics, and test how providing psychological and motivational support for university enrollees may influence the elimination of this type of fear. The objectives of the study were as follows:

- Study and explain the fear of starting training and highlight its significant features
- Form a psychometric tool (questionnaire and test) to analyze the level of fear of starting training in adults
- Create the methodology rising motivation to learn and providing psychological support for first-year students before starting university and test its effectiveness.

The novelty of this study is explained by an almost complete absence of academic research on the features of stress and fear management in students before the beginning of the educational process.

For the study purposes, the following *null hypotheses* were formulated to be further tested:

*H*_0_: There are no statistically significant differences between the levels of fear of starting training before and after the educational process began.

*H*_1_: There are no statistically significant differences between the level of fear of starting training in the group receiving no special support and the group receiving such support.

## Materials and methods

### Participants

The study was conducted at two universities simultaneously (one of them is located in the Russian Federation and one – in China). Its sample included a total of 536 individuals who had just entered these institutions (268 people from each university) and were ready to begin their studies. For both universities, experimental and control groups of approximately equal size (134 people) and number of males and females were created. The participants were sampled by random sampling among the newcomers. Their demographic distribution is presented in detail in Table 1. The age range was from 18 to 21 years old. An equal number of respondents was chosen to ensure that the Student’s *t*-test was applied with an equal number of items for statistical analysis.

**Table 1:** Sample demographics.

	Experimental group		Control group	
	Male	Female	Male	Female
Russian	68	66	67	67
Chinese	69	65	66	68

No demographic criteria for participant selection were controlled for in this sample; the distribution of demographic categories was entirely random. Considering the number of applicants to both universities participating in the study as the general population, the acceptable sampling error for the entire population of participants was 3.42 (3.21 for the Russian university and 3.71 for the Chinese university). Hence, the sample can be considered statistically representative of the group of university entrants.

The selection of prospective students as the study group is explained by the fact that university entrance represents the most substantial arousal of fear of starting a degree compared to other similar triggers. Based on the sources studied and available empirical research (Bell and Williams, 2006; Chetty and van der Westhuizen, 2013; Alt, 2015), it was suggested that fear of starting university should be markedly lower at the beginning of the next academic year if the student is already familiar with student life and the pressure of academic demands. Likewise, the fear of starting another course or subject should be lower when such an experience is already gained (Conrad, 2002; Corral-Lage et al., 2020).

### Research design

#### Development of the modified Kessler psychological distress scale (K10)

Investigation of the fear of starting training was decided to be conducted using a modified version of the Kessler psychological distress scale (K10). The K10 test was chosen as one of the most commonly employed in clinical practice and psychometric studies of common fears. Apart from this, it is also recommended by national clinical associations (Andrews and Slade, 2001; Anderson et al., 2013). Since the K10 test focuses on identifying general distress and its depth and is adapted to diagnose the need for intervention by a mental health professional, it was modified to meet the needs and goals of this study. In sum, the K10 contains 10 questions, each of which is rated on a 5-point Likert scale. The maximum number of points (50) corresponds to the strongest state of fear and most severe destructive distress. The normal level is considered to be 20 points, and the intervention by a specialized psychologist is recommended at 24 points or higher. The last issue to mention is that the K10 test is to be passed independently.

As was indicated earlier, the K10 test was somehow modified for the study purposes. In particular, a 30-day self-observation period for the interviewee, on which the self-assessment questions are based, was shortened to 10 days. The wording of the questions related to the general state of fear without specifying its source or cause was modified to pinpoint the source of the stress. Thus, for example, Question 1: “During the last 30 days, about how often did you feel tired out for no good reason?” was replaced by the modified version: “During the last 10 days, about how often did you feel tired due to waiting for the start of training?” Other questions were changed similarly.

#### Checking the time for stress from waiting for the training start to decay

In the decision to shorten a 30-day self-observation period to 10 days we relied on already available data from a number of empirical studies in which the level of fear induced by stress from waiting for a certain event tends to fall over approximately 14–10 days (Dixon and Pelliccione, 2004; Mineka and Sutton, 2006; Pleše et al., 2018). However, these data cannot be considered wholly accurate and valid because they do not make allowances for subjects’ personal characteristics, the presence of comorbid stressors, illnesses, psychological dispositions, or the level of background accumulated stress. Consequently, an additional examination was decided to be conducted. This examination presupposed administering the K10 test using the wording of 10 days instead of 30 days in each question to a randomly selected sample of 98 applicants of a Russian university who were preparing to enter their first study year. In sum, this testing was conducted four times: 30, 20, and 10 days before the start of the course (September 1) and immediately after the completion of academic classes on the first day of study. The results obtained for each of the four consecutive tests were compared in pairs using the Student’s *t*-test (Table 1).

**Figure 1:**
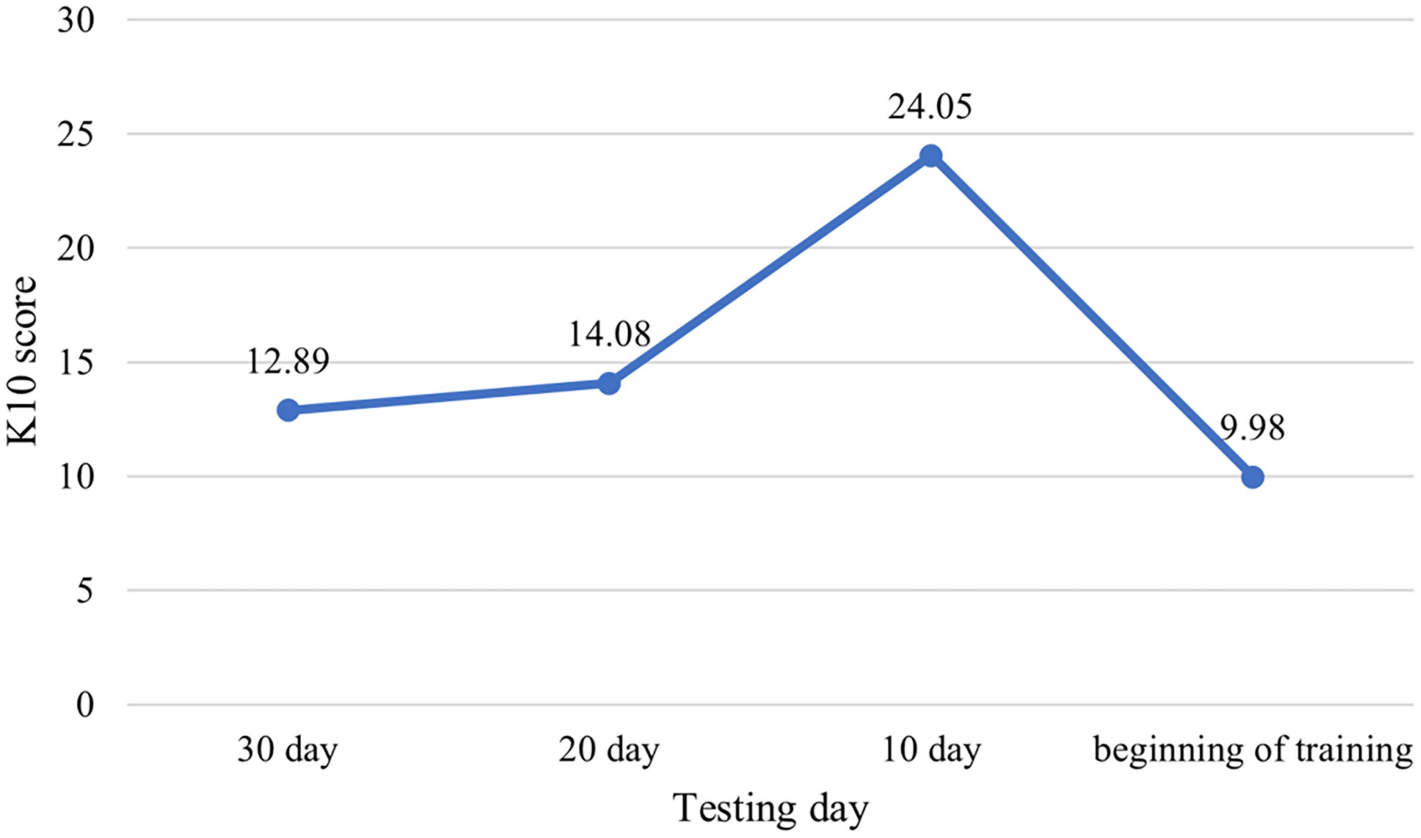
K10 pre-examination outcomes.

Figure 1 below gives a graphical representation of K10 scores obtained during each testing session. As can be seen, there is a sharp spike in fears at a time that tentatively coincides with the 10-day term before the study began. Since testing was not conducted daily, it was impossible to record more precise timing of this effect and provide a basis for broader generalizations. Though, the collected values do confirm the significance of the 10-day term in determining the strength of fear of starting training in the proposed version of the K10 test.

Values presented in Table 2 allow the inference that only the 10-day term test data have statistically significant differences from the results of the other three tests. Hence, it is the 10-day term that is most optimal for use in the modified K10 test.

**Table 2:** *p*-values for paired Student’s *t*-test comparing the K10 results.

	Mean	SD	*t*-test	Cohen’s *d*	*p*-value
*30 day to 20 day*					
30 day	22.18	2.03			
20 day	18.27	1.27	1.963	2.3092	0.3441
*30 day to 10 day*					
30 day	22.18	2.03			
10 day	14.34	1.18	1.9622	4.722	0.0341
*30 day to beginning of training*					
30 day	22.18	2.03			
Beginning of training	9.57	1.56	1.9621	6.9656	0.1249
*20 day to 10 day*					
20 day	18.27	1.27			
10 day	14.34	1.18	1.962	3.206	0.0245
*20 day to beginning of training*					
20 day	18.27	1.27			
Beginning of training	9.57	1.56	1.9622	6.1164	0.2569
*10 day to beginning of training*					
10 day	14.34	1.18			
Beginning of training	9.57	1.56	1.962	3.4487	0.0012

To examine the validity of the modified version of the K10 test, Cronbach’s alpha (*α*) was calculated. It enabled comparing data from the two main tests conducted as part of the main study. Insofar as the obtained value equaled 0.874, the high validity of the modified test was confirmed. The reliability of the test was verified using the test-retest method. The retest was conducted before the beginning of the second semester during the vacation period and 6 months after the experiment described. Its outcomes were compared by calculating Pearson’s correlation coefficient when analyzing test scores of the same participants. The correlation level corresponded to *r* = 0.9807 for maximally similar test conditions (the content and context of stress that causes the fear of starting training).

#### Main experiment to investigate the fear of starting university training

The main experiment was conducted simultaneously for Chinese and Russian groups of participants under the same conditions. The first test was administered 10 days before the study begun, and the second test – 2 days after the classes started. No special intervention was used in control groups (CGs) of both universities. In turn, the students from experimental groups (EGs) received specialized assistance directed on training motivation increase and psychological support already from the 10-day term prior to the start of classes (the first of the tests was completed before the intervention).

### Data analysis

The K10 testing outcomes obtained in all groups were checked for normal distribution using the *χ*^2^ test. Once it was determined that the survey data obey the normal distribution law, null hypotheses of the study were tested under the Student’s *t*-test for the following series of data:

1. EG and CG results were compared to determine the presence of statistically significant differences that could confirm the effectiveness of the impact of psychological and motivational support
2. CG results collected before and after the beginning of the training were compared to check for statistically significant differences in the feeling of fear before and after the educational process started
3. EG results collected before and after the beginning of the training were examined and compared with the study data to see if the change in the level of fear after the start of classes differed due to the provision of psychological support and motivation-rising tactics.

### Intervention

As part of implementing the measures entailing qualified psychological support and increasing motivation to start learning, students were offered a number of interventions to help alleviate various aspects of the stress from waiting for the educational process to begin:

1. A detailed introduction to the future learning process, the subjects offered, and the teaching methods used at each university.
2. Conducting a real and/or virtual university tour with an explanation of the peculiarities of everyday organization of learning, communication, movement between classrooms, and students’ leisure time.
3. Daily interaction with 1–5 years students for 1 h or more directly at the university campus, facilities, and classrooms for 10 days.
4. Familiarization with real examples of how first-years completed assignments with comments from their professors and students themselves (these materials were provided by freshmen willing to offer their assistance during the experiment on an anonymous basis).
5. More active and prolonged communication with family and friends during the 10 days prior to the training start. Participants were asked to estimate the average time they devoted to such communication and increase the amount of interaction with their relatives by 30–50% over the 10 days before the start of the course. All types of communication were included: direct verbal communication, doing household chores together, shared hobbies or sports or other games, walking or shopping together, etc.
6. Devoting at least 1 h to hobbies or favorite activities of any type during 10 days.
7. Active involvement in physical activity of any suitable or familiar type (fitness, yoga, various sports, swimming, brisk walking, etc.) for at least 1 h per day.

An agreement was reached with the participants to keep an immediate online diary in which they noted their activities associated with this training program immediately during or after this activity. This could be done with a single click on a mobile device in a special form previously created in the cloud service. The researchers also received confirmation of the activity of the participants from their relatives or friends - observers, who also noted a similar form. The simplicity and ease of performing such control, as well as the absence of pressure or commitment from the participants and their assistants, give a high confidence that the activities were actually carried out and celebrated. The adherence of participants to the program can be confirmed by the fact that the marks of the participants and the assistants who supervised them differed only in <6% of cases, and only <4% of the activities were missed for all participants.

### Statistical processing

To analyze the statistical data obtained during the questionnaire test, SPSS 6.0 statistical processing package was used. Data visualization was performed in Microsoft Excel 2019.

### Ethical issues

All respondents were provided with a detailed explanation of the nature and focus of the study and gave their written voluntary consent to participate. No personal information about the persons enrolled was collected, stored, or analyzed during the investigation. Participants’ academic performance was not influenced in any way by the study course or by their participation in it.

### Research limitation

This research did not consider gender or other demographic differences as factors influencing the experience of fear of starting training. It also did not take into account the presence of background stress or personal psychological history of the participants or the availability of any physiological or psychological impairment that might affect fear of learning. Another point of concern that is to be noted is that there could be participants’ subjective evaluative factors that might influence the assessment results. As a consequence, there was a possibility that the state of fear to start learning might be attributed to another experienced stress and not the one addressed in this paper.

## Results

**Figure 2:**
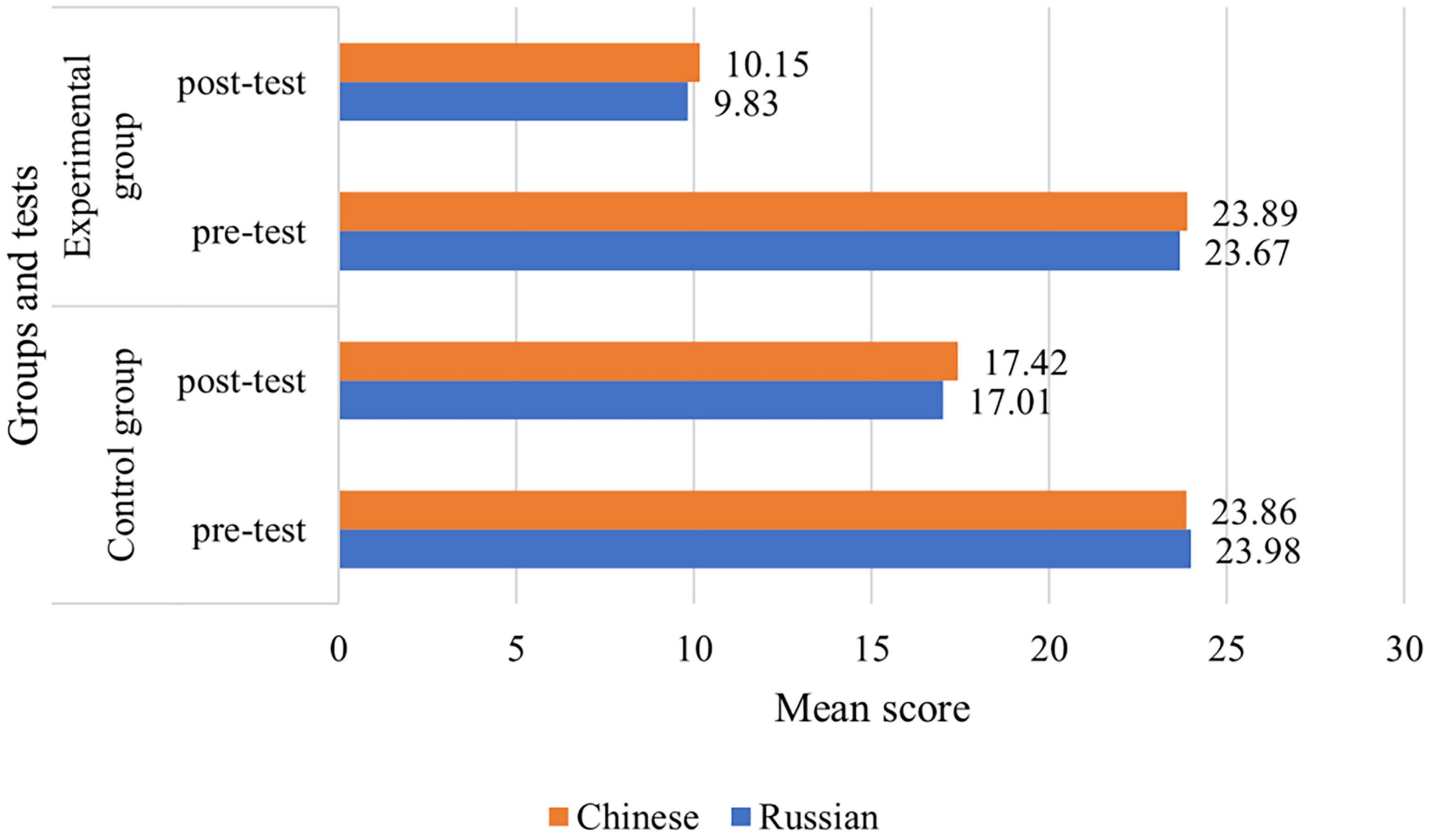
Pre- and post-test results separated by level of fear of starting training, %.

To simplify further statistical data presentation, the results for the Russian and Chinese groups were combined together. This was done as a result of the fact that the performed Student’s *t*-tests revealed no statistically significant differences between the Chinese and Russian samples (the lowest p-value for all tests between the Chinese and Russian groups was *p* = 0.2385, which designates clearly the absence of significant differences). In view of this, hereafter, “CG” should be understood here as CGs of Russian and Chinese universities simultaneously. The same applies to “EG” and the term “groups.” This is true for all data except Figure 2, where the national component was indicated for the sake of clarity.

**Figure 3:**
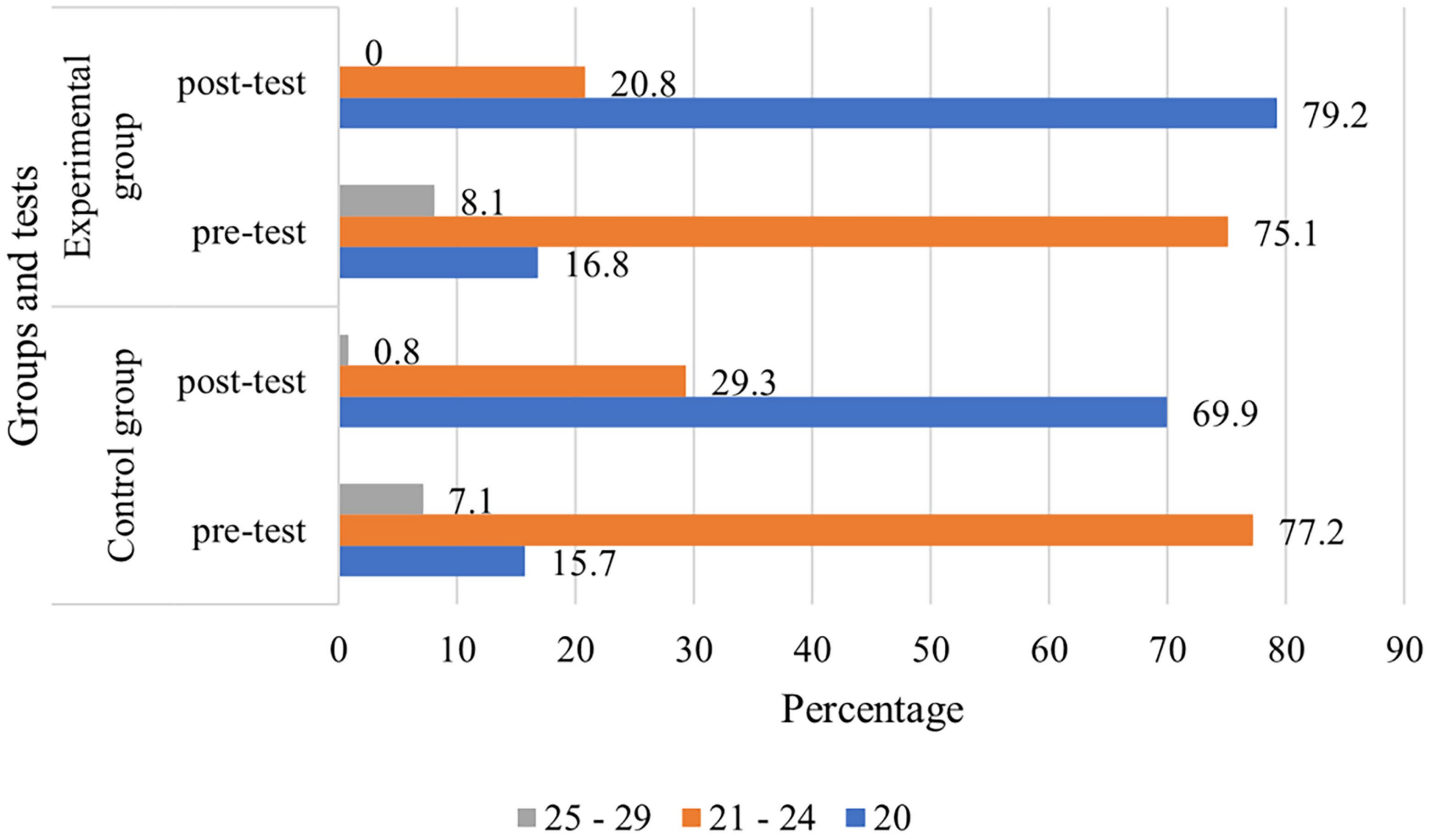
Pre- and post-testing results by group.

In sum, the results of the carried out testing demonstrate a marked reduction in fear and stress after the start of training for both groups of both universities (Figures 2, 3). Figure 3 depicts the grouping of all test participants according to the interpretation of the K10 results. Here, a test score of <20 (Subgroup 1) indicates a normal condition that does not require intervention or correction. A score of 21–24 (Subgroup 2) indicates the presence of a mild problem with which the individual is able to cope independently. At the same time, a test score of 25–29 (Subgroup 3) represents a moderately severe fear-based disorder that may already require intervention and assistance. As shown in the Figure 3 below, Subgroup 3 with the highest level of fear is the most significant as it includes 7.1% of CG and 8.1% of EG participants (according to the results of the pre-test performed 10 days before the study). During the post-test (conducted 2 days after the course started), the volume of this subgroup dropped to zero in EG and to minimal values (0.8%) in CG (Figure 3). This may indicate that such a high level of fear of starting training in Subgroup 3 respondents is due to their personal psychological and behavioral context or features.

Most meaningful in terms of the research topic is the change in the ratio of subgroups by the assessed EG condition. The ratio of those with no disturbances to those having them (Subgroup 2, 21–24 points) in the CG was virtually the same, only the overall average score decreased. In the EG, in turn, this ratio shifted radically: those who felt noticeable tension from fear became four times fewer than those who felt quite well (20.8% and 79.2%, respectively). The rational explanation for this difference is the intervention of specialists giving support and rising students’ motivation prior to the start of training.

As evidenced in Figure 2, post-test scores of EG and CG improved noticeably, even though in EG, the distress level due to fear of future learning was by almost a third lower than in CG (10.15 points in the Chinese and 9.8 points in the Russian EGs vs. 17.42 and 17.01 in CGs, respectively). This observation demonstrates the already described effect of a significant reduction in fear after the start of training connected with the absence of expectation and encountering with the reality with which one can cope on his/her own.

The test results’ comparison presented in Table 3 indicates that the differences between the pre-and post-testing outcomes collected for EG and CG are equally statistically significant (*p* amounts to 0.0287 for EG and 0.0309 for CG). Thus, it can be argued that there is a notable decrease in the level of fear of starting university training after the classes began. On the other hand, the fear was not eliminated, although it fell to a level acceptable in terms of clinical psychology, which does not suggest any mental problems.

**Table 3:** Student’s *t*-test results for the values of the modified K10 test.

	Experimental group					Control group				
	Mean	SD	*t*-test	Cohen’s *d*	*p*-value	Mean	SD	*t*-test	Cohen’s *d*	*p*-value
Pre-test	23.17	2.18				23.88	2.08			
Post-test	9.91	1.31	1.9691	7.3732	0.0287	17.17	2.29	1.9692	3.0673	0.0309

The results of the Student’s *t*-test for EG and CG’s post-test values (Table 4) also show statistically significant differences.

**Table 4:** Student’s *t*-test results for EG and CG’s post-test values of the modified K10 test.

	Mean	SD	*t*-test	Cohen’s *d*	*p*-value
Experimental group	9.91	1.31			
Control group	17.17	2.29	1.969	3.8917	0.0181

This testifies to the fact that a better state of affairs in EG (Figures 1, 3) is not a manifestation of random statistical fluctuations, and the proposed psychological and motivational support significantly reduced the fear of starting training.

According to the data obtained, both formulated *null hypotheses* should be rejected as there are statistically significant differences both between the level of fear associated with the beginning of training before and after the study course started (*H_0_*) and between the level of fear reduction in the group receiving specialized psychological support and the group not receiving it (*H_1_*).

Despite the fact that the average results of EG and CG at both universities surveyed were at the level of sustained psychological health, the difference in the well-being of students who received support is clearly visible. The improvement of their general psychological state can be partially identified by the example of a group of students who were in a state of insignificant but already recordable emotional disorder (Figure 3). While in the case of the CG, the number of such students decreased, in EG, there were none of them at all.

More indicative of the impact of student support and motivation techniques might be a test on subsequent and repeated instances of stress, such as the beginning of future training courses or taking up classes that are expected to be more difficult in their academic mastery. Furthermore, it may be reasonably assumed that those students who have already experienced support will show lower levels of fear of starting a course than those who have not received any help. Examination of this point will become a topic of one of the further studies.

## Discussion

Today’s reality is that academic fear research rarely concentrates on the fear of starting to learn. Instead, it focuses on the fear of starting something new or fear of success in the area of learning (Garcia-Ruffin, 2003; Bell and Williams, 2006). Consequently, when analyzing the findings of this study, it is necessary to draw on the data on fears specific to the learning process in general and the obvious reactions related to the beginning of learning.

Most representative studies in the field were conducted in programming instruction (Conrad, 2002; Chetty and van der Westhuizen, 2013). They identified a range of fears and forms of anxiety tested separately and analyzed in factor relationships between them. Their results show many effects similar to those reported in the current study. In particular, the severest manifestations of fear always accompany the period before learning or before deep learning immersion (Keckler, 2018). Unfortunately, most research works fail to conduct continuous multi-step testing that could reveal the dynamics of fear or anxiety formation and reduction, so it is difficult to isolate any particular predictors of changes in fear in learners. However, it is known that fear and anxiety levels decrease after students begin learning, during mastering subjects, and after gaining initial knowledge and confidence in their ability to cope at least minimally (Bach and Melinscak, 2020). And this is fully in line with the findings of the current study.

Students’ individual experiences of starting the training process play a critical role in fear reduction and rise of the motivation to continue learning (Dixon and Pelliccione, 2004). Students who cannot cope with the pressures of the fear or anxiety are more likely to be in the group of those refusing to continue studies (Alt, 2015). Obviously, testing with K10 could demonstrate distress indicators in this group of students that are consistent with an average stress level. However, as it was shown, it is the use of support and motivation means that provide the opportunity to reduce the number of participants at risk critically. Such conclusions are indirectly supported by judgments from other empirical studies addressing learning-related fears (Dias et al., 2016; Carneiro et al., 2018).

Some researchers associate fears accompanying the process of training with the use of specialized teaching methods, such as problem-based learning, for instance. These methods require a greater degree of proactive participation of students as well as independent search and solution of real problems. Though, despite their effectiveness, these requirements contribute to a substantial increase in fear of failure, fear of social non-recognition, and fear of one’s own incompetence (Correia and Marques-Pinto, 2016; Corral-Lage et al., 2020; Csehi et al., 2020). It must be admitted that these particular types of fears can be seen as components of a more complex experience, defined in this paper as fear of starting training. The intensive forms of learning may cause different dynamics of the activity of fears connected with the learning process. They may naturally decrease at the beginning of training and then increase sharply and even stabilize when the student is drawn into problem-oriented learning, STEM education, case-based learning, etc. In parallel, empirical studies prove that even when a student consistently copes with this type of sustained and repetitive stress, the level of fear and anxiety can remain consistently high and gradually lead to an accumulation of distress and burnout (Corral-Lage et al., 2020). Therefore, it is imperative to accompany and support students when applying techniques intensifying knowledge acquisition.

Empirical evidence shows that the measures directed on preliminary adaptation are more helpful than subsequent support for students (Pleše et al., 2018). These results are generally in line with those obtained in this study because it is preconditioning, which includes intensive acquaintance with academic life and requirements, that contributes to a critical reduction in fear.

## Conclusion

The fact that fears associated with learning can affect student and school activities, in particular, determine academic performance, personal development, and relationships with others, is not new these days. However, it is quite unexpected that the complex phenomenon of fear associated with the start of training is investigated poorly. This paper focused on determining the distinguishing features of this type of fear and investigated whether motivational and psychological support can raise first-years’ desire to learn and reduce the stress from the study beginning. In the course of the research process, two groups of a total of 536 future first-year students from Russian and Chinese universities were involved. Both groups were equally divided into control and EGs, though representatives of the second received psychological and motivational support. To solve the problem of psychometric assessment of the level of fear, the modified K10 test was created. Testing was done 10 days before and 2 days after the start of university training. Pre- and post-test results in all groups were comparatively assessed using the Student’s *t*-test. The collected values confirmed statistically significant differences related to the decrease in the level of fear in all groups before and after university classes started. This allows one to deem the proposed support methodology quite effective.

In practice, the collected findings can be widely applied without additional costs for the initial adaptation of new students and stable improvement of their future academic results. Further research will be concentrated on conducting regular sequential tests to determine the dynamics of changes in the level of fear of learning in students who have started their university studies. Apart from this, it is planned to define the influence of various factors that may act as predictors of fear of starting training and recognize the features of this type of fear in groups with different demographic characteristics.

## Data availability statement

The original contributions presented in the study are included in the article/supplementary material, further inquiries can be directed to the corresponding author.

## Ethics statement

Ethical review and approval was not required for the study on human participants in accordance with the local legislation and institutional requirements. Written informed consent from the (patients/participants OR patients/participants legal guardian/next of kin) was not required to participate in this study in accordance with the national legislation and the institutional requirements.

## Author contributions

TL, VP, IS, NI, and SS contributed to the study conception and design. Material preparation, data collection and analysis were performed by TL, VP, IS, and NI. The first draft of the manuscript was written by SS. All authors read and approved the final manuscript.

## Funding

NI has been supported by the Kazan Federal University Strategic Academic Leadership Program.

## Conflict of interest

The authors declare that the research was conducted in the absence of any commercial or financial relationships that could be construed as a potential conflict of interest.

## Publisher’s note

All claims expressed in this article are solely those of the authors and do not necessarily represent those of their affiliated organizations, or those of the publisher, the editors and the reviewers. Any product that may be evaluated in this article, or claim that may be made by its manufacturer, is not guaranteed or endorsed by the publisher.
